# EVALI, anti-vaping advertising, quit attempts, and susceptibility: insights into the 2019 inflection point for U.S. adolescent vaping from California data

**DOI:** 10.1186/s12889-026-27209-3

**Published:** 2026-04-02

**Authors:** Jijiang Wang, Anthony C. Gamst, Yue-Lin Zhuang, Shu-Hong Zhu

**Affiliations:** 1 Herbert Wertheim School of Public Health and Human Longevity Science, University of California, San Diego, 9500 Gilman Dr., La Jolla, San Diego, CA 92093-0905 USA; 2 Department of Mathematics, University of California, San Diego, 9500 Gilman Dr., La Jolla, San Diego, CA 92093 USA; 3 Moores Cancer Center, University of California, San Diego, 9500 Gilman Dr., La Jolla, San Diego, CA 92093 USA

**Keywords:** Adolescent, Vaping, Quit attempt, Intention to quit, Media campaign, EVALI, Susceptibility

## Abstract

**Background:**

Adolescent vaping prevalence in the U.S. surged dramatically from 2017 to 2019, then declined significantly, making 2019 an inflection point. Anti-vaping advertising and media reporting on e-cigarette or vaping use-associated lung injury (EVALI) may have contributed to the decline. This study examined whether anti-vaping advertising exposure and EVALI awareness were associated with adolescents’ quit attempts, intentions to quit, and susceptibility to future vaping.

**Methods:**

Data from the 2017–2018 (*N* = 117 757) and 2019–2020 (*N* = 143 565) California Student Tobacco Survey were analyzed. Participants’ quit attempts, intentions to quit, and susceptibility to future vaping in the two periods were compared. Multivariable logistic regressions were used to examine the effects of anti-vaping advertising exposure and EVALI awareness on these three dimensions.

**Results:**

Compared to 2017–2018, middle and high school students who currently vaped in 2019–2020 had higher rates of quit attempts (53.2% vs. 28.8%, *p* < 0.001) and intentions to quit (79.1% vs. 56.9%, *p* < 0.001). Susceptibility to future vaping among those who never vaped was lower in 2019–2020 than in 2017–2018 (25.7% vs. 30.3%, *p* < 0.001). Exposure to anti-vaping advertising and awareness of EVALI were significantly associated with higher rates of attempting quitting and intentions to quit. EVALI awareness was negatively associated with susceptibility to future vaping.

**Conclusions:**

EVALI awareness and exposure to anti-vaping advertising were associated with positive changes in adolescents’ vaping-related attitudes and behavior. A substantial increase in quitting activity among those currently vaping and a decrease in susceptibility to future vaping among those who never vaped likely contributed to the decline in adolescent vaping prevalence since 2019. Future interventions should leverage paid and earned media to facilitate a continued decline in youth tobacco use.

**Supplementary Information:**

The online version contains supplementary material available at 10.1186/s12889-026-27209-3.

## Background

**Fig. 1:**
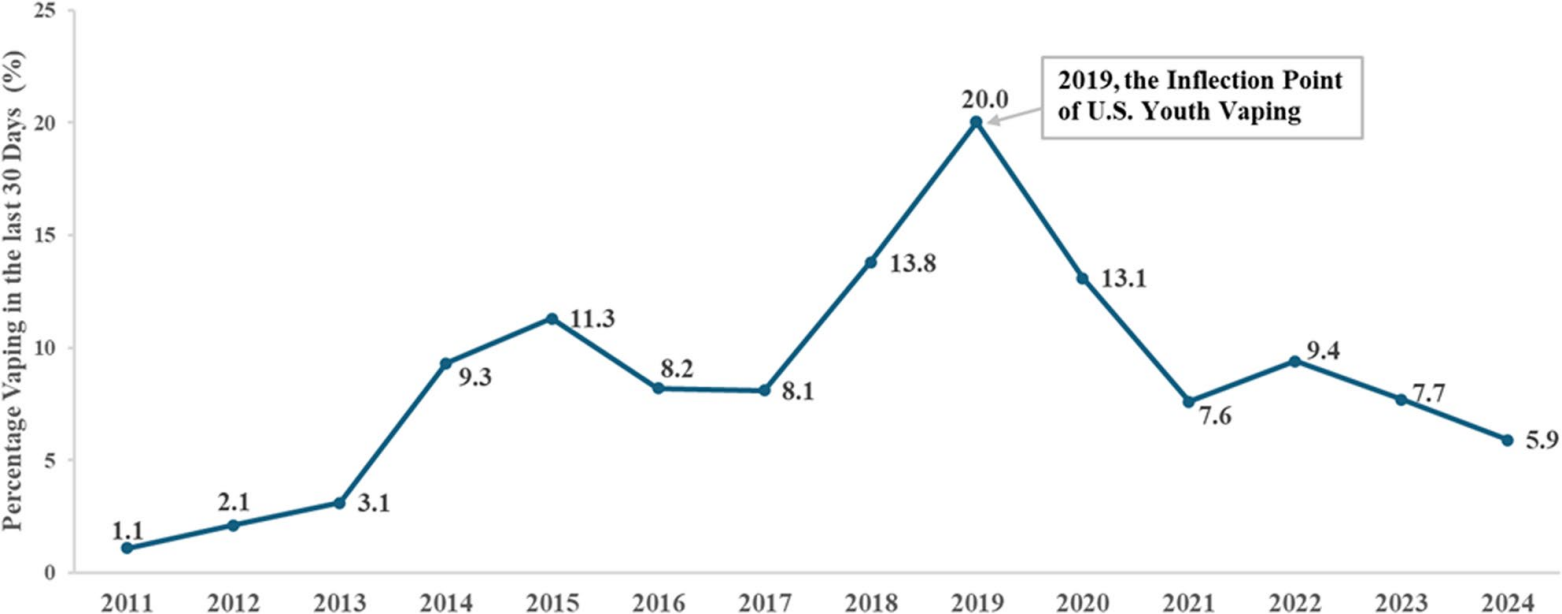
Vaping prevalence among middle and high school students in the U.S., National Youth Tobacco Survey [1, 2].

When the National Youth Tobacco Survey began monitoring the prevalence of vaping among U.S. adolescents in 2011, the rate was 1.1% (Fig. 1) [1, 2]. Thereafter, it generally increased, with some fluctuations, reaching 8.1% in 2017. From 2017 to 2019, the rate surged to 20.0% [1]. However, it decreased significantly to 13.3% the following year and fell further to 5.9% in 2024 [1, 2]. Several studies have investigated the reasons for the dramatic increase in youth vaping from 2017 to 2019 [3–5]. This study sought to understand the reasons for the decline since 2019.

Some have suggested that a changing media environment regarding vaping helped make 2019 an inflection point [6–8]. Two factors in this regard are especially noteworthy. One was aggressive anti-vaping advertising targeting young people beginning in 2018 and then expanded in 2019 [9–12]. The other was extensive media reporting on an outbreak of e-cigarette or vaping product use-associated lung injury (EVALI), beginning in July 2019 and lasting into 2020 [8]. These two factors may have cast vaping in a sufficiently negative light to affect adolescents’ perceptions of vaping [6–8]. 

Adolescents in the U.S. may have encountered anti-vaping ads from multiple sources. The Food and Drug Administration (FDA) launched a campaign in 2018 to educate youth about the dangers of vaping. Modeled on an earlier FDA effort to prevent youth smoking, the campaign began digitally and expanded to television in 2019 [11]. Also in 2018, the Truth Initiative launched a campaign to increase awareness among young people that vaping, while safer than smoking, is not safe [12]. Many states also conducted anti-vaping campaigns. For example, California launched a campaign in 2018 to increase awareness of how the tobacco industry uses flavored vapes to hook kids; the campaign was expanded in 2019 to respond to EVALI [9, 10]. The campaigns achieved substantial reach among adolescents and impacted their beliefs and behavior [13–15]. 

Almost contemporaneous with these campaigns, the EVALI outbreak was unfolding. It was associated with over 2800 hospitalizations and 68 deaths, many among young people, and media coverage was massive. The New York Times alone published 141 articles on EVALI in the second half of 2019 [16]. From July 2019 to March 2020, nearly 20 000 articles on EVALI appeared online [8]. The outbreak’s cause, vitamin E acetate in certain illicit tetrahydrocannabinol (THC) vape products, was not initially identified [17]. Therefore, most media reports discussed EVALI in the context of vaping in general; nearly half of the reports mentioned youth vaping as a concern [16, 18]. Awareness of EVALI became widespread among young people. Three quarters of adolescents surveyed in California reported having heard about EVALI; most said they first heard about it from the media and most incorrectly believed nicotine to be the cause [8]. These data suggest that news reports on EVALI likely had a significant impact on adolescent perceptions of vaping.

For an intervention to reduce vaping prevalence, it must increase quitting or reduce initiation. If the media factors described above helped make 2019 an inflection point in adolescent vaping prevalence [6–8], either the quit attempt rate should be higher in 2019 than in preceding years or non-vapers’susceptibility to vaping in the future should be lower, or both. Such results, if observed, would not prove that media factors caused the decrease in adolescent vaping prevalence, but would be consistent with the hypothesis that they helped reverse the upward trend. Moreover, if media factors influenced quit attempts or susceptibility to future vaping, a regression analysis may detect correlations between media exposures and these vaping-related outcomes.

Unfortunately, nationally representative surveys have measured adolescent exposure to anti-vaping advertising but not awareness of EVALI. One statewide survey that measured both was the 2019–2020 California Student Tobacco Survey (CSTS) [19]. The preceding 2017–2018 CSTS was conducted from September 2017 to June 2018, before the launch of national anti-vaping advertising campaigns [20], whereas the 2019–2020 CSTS was conducted well after the launch of the campaigns (September 2019 – March 2020), following peak EVALI media coverage and before COVID-19-related school closures [19]. Both surveys measured exposure to anti-vaping advertising and various cognitive and behavioral variables related to vaping. In addition, the 2019–2020 CSTS measured awareness of EVALI. The timing of the two surveys makes it feasible to examine the possible effects of these media factors on adolescents.

Previous studies analyzing data from these two CSTS cycles showed that adolescents’ exposure to anti-vaping advertising increased significantly from 2017–2018 to 2019–2020 [7], that most respondents knew about EVALI when taking the 2019–2020 survey [8], and that those who knew about EVALI perceived vaping as riskier than those who did not [8]. The current study used these two CSTS cycles to evaluate the effects of media factors on vaping-related outcomes among California students, to illuminate factors that may have contributed to the change in the national trend in adolescent vaping. Specifically, it sought to determine whether anti-vaping advertising and EVALI awareness were associated with higher odds of quit attempts and quitting intentions among current vape users and lower odds of susceptibility to future vaping among those who had never used vapes.

First, respondents to the two surveys were compared regarding current vape users’ rates of making a quit attempt and intentions to quit, and never-vapers’ susceptibility to future vaping [21]. Second, we examined the relationship between two key media factors—exposure to anti-vaping advertising (the results of paid media) and awareness of EVALI (the results of earned media)— and survey respondents’ quit attempts, quitting intentions, and susceptibility to future vaping. For the second step, multiple logistic regressions were employed to examine the effects of paid and earned media simultaneously.

## Methods

### Data source and participants

Data in this study came from two cycles of the CSTS, a tobacco use survey that was conducted biennially among middle and high school students in California. It employed a probability sample of schools designed to represent students in grades 8, 10, and 12 statewide. Surveys were administered via an online questionnaire assessing tobacco use patterns, quitting behaviors, knowledge, attitudes, and other factors such as tobacco-related media exposure. Detailed descriptions of the survey designs can be found elsewhere [19, 20]. 

CSTS cycles conducted during the 2017–2018 and 2019–2020 school years included 151 404 students and 162 675 students, respectively. For the present study, only students who were current vape users (i.e., had vaped in the last 30 days) or who had never vaped were included. Former vape users were excluded because they were not assessed for recent quitting behaviors or susceptibility to future vaping. Moreover, only students who answered all questions on vaping status, quit attempts, intentions to quit, and susceptibility to future vaping were included. This resulted in effective samples of 11 966 current vape users and 105 791 never-vapers in the 2017–2018 CSTS, and 11 767 current vape users and 131 798 never-vapers in the 2019–2020 CSTS.

### Measures

#### Exposure variables

##### Vaping-related advertising exposure

Exposure to vaping-related ads was assessed with two questions. The first was, “In the last 30 days, have you seen ads that were either promoting or discouraging the use of … e-cigarettes (or vapes)?” Those who had seen such ads were asked, “Did the e-cigarette (or vape) ads you saw …” with the options, “Mostly promote their use,” “Mostly discourage their use,” “Neither promote nor discourage their use,” and “I do not know.” Respondents who chose “mostly discourage their use” were considered exposed to anti-vaping advertising, while all others were considered not exposed.

##### EVALI awareness

EVALI awareness in 2019–2020 was assessed with the question, “Have you heard about people getting sick or even dying from using vapes?” (Yes/No). This question was added to the 2019–2020 CSTS on September 29, 2019, when information about EVALI became widespread. Similar to other surveys on EVALI [22–25], the question in this survey did not explicitly name EVALI but described the severe vaping-related illness during the EVALI outbreak period, which coincided with the time the survey was fielded. A total of 2353 students (1.6%) completed the survey before the EVALI question was added.

#### Outcome variables

##### Quit attempts

Participants who vaped in the last 30 days were asked, “In the last 12 months, did you try to quit using e-cigarettes?” Those who responded yes were considered to have attempted quitting.

##### Intentions to quit vaping

Students who vaped in the last 30 days were also asked, “Do you plan to quit using e-cigarettes?” Those who answered, “I already quit,” “Yes, I plan to quit in the next month,” or “Yes, I plan to quit sometime, but not in the next month” were considered to have quitting intentions. Those who answered, “No, I do not plan to quit” were considered not to have quitting intentions. In 2017–2018, the question also included the option, “I prefer not to answer.” Students who selected this were excluded from the analysis. In the 2019-20 survey, the term “nicotine vapes (with or without flavor)” was used to replace “e-cigarettes” in the questions to reflect the change in the terminology used by the adolescent population.

##### Susceptibility to future vaping

Students who had never used vapes were asked, “If one of your BEST FRIENDS offered you an e-cigarette, would you use it?”, an item selected from the Expanded Susceptibility to Smoke Index [21]. Those who answered, “Definitely yes,” “Probably yes,” or “Probably not” were considered susceptible to future vaping. Only those who answered, “Definitely not” were considered not susceptible. In 2017–2018, the question also included the option, “I prefer not to answer.” Students who selected this were excluded from the analysis.

#### Covariates

##### Sociodemographics

Grade level served as a proxy measure for age, with grades 8, 10, and 12 corresponding roughly to ages 13, 15, and 17, respectively. For gender identity, students were categorized as male, female, other gender, or declined to state/missing. For race/ethnicity, students were categorized as Hispanic, non-Hispanic (NH) White, NH Black, NH Asian, NH multiracial, NH other, or declined to state/missing. Parental education, defined as having at least one parent who was a college graduate, served as a proxy measure for socioeconomic status.

##### Mental health

Students rated their mental health as “Excellent,” “Very good,” “Good,” “Fair,” or “Poor.” Responses were collapsed into two categories: good-to-excellent and fair-to-poor.

##### Other tobacco-related behaviors

Students were also categorized by whether anyone had offered them a vape in the last 30 days, whether they had used vapes on 10 or more of the last 30 days, and whether they had used any other tobacco products in that timeframe. Vaping frequency was dichotomized using a ≥ 10-day cutoff in the past 30 days to distinguish higher-frequency use from lower-frequency use [26–28]. Students who reported using any of the following products in the past 30 days—cigarettes, big cigars, little cigars or cigarillos, hookah, smokeless tobacco, or heated tobacco products—were classified as current users of other tobacco products.

### Statistical analysis

Estimates for quit attempts, intentions to quit, and susceptibility to future vaping were calculated with 95% confidence intervals (CI) [29]. Confidence intervals for the estimates were used for between-cycle and between-group comparisons. Comparisons for estimates with overlapping CIs were tested with the Wald Chi-Square Test. Multivariable logistic regression was used to assess, for current vape users in the 2019–2020 sample, the associations between quit attempts and anti-vaping advertising exposure, and between quit attempts and EVALI awareness, controlling for the effects of covariates, including demographic variables, mental health status, frequency of vaping, use of other tobacco products, and being offered vapes. To assess the unique contribution of advertising exposure and EVALI awareness, we examined the coefficients for each of them by including one measure only or including both measures simultaneously in the regression models. They yielded stable coefficients under both conditions and there was no sign reversal. Therefore, we included both exposure variables in the same logistic regression model. Parallel analyses were conducted for intentions to quit vaping among current vape users and susceptibility to future vaping among never-vapers. Sample weights reflecting the sampling process and the distribution of students in California were applied for all analyses. Complex survey procedures were used to account for design effects related to regional strata and clustering within schools. Pairwise deletion of cases containing missing data was applied for all descriptive analyses. Cases with at least one missing data point were removed in the multivariable logistic regressions (including 7.0% in the current vape users sample and 3.5% in the never-vapers sample; 80% of missing data points were due to not being asked or not responding to the EVALI question). Data were analyzed in 2024 − 2025 using SAS 9.4.

## Results

Table 1 shows the weighted distribution of current vape users and never-vapers by demographic characteristics. Never-vapers shared a similar gender and grade distribution in the two surveys, but in the later survey current vape users were more likely to be female (*p* < 0.001). Because the race/ethnicity questions in 2017–2018 included the option “I prefer not to answer” and those in 2019–2020 did not, race/ethnicity data were analyzed in two ways: once with students who chose not to answer and missing data included, and again with those students excluded. When excluding those who did not report their race/ethnicity, the distribution was not significantly different between the two surveys. Table 1 also includes vaping frequency among students who vaped in the past 30 days. The proportion of vaping on 10 or more days did not differ significantly between 2017–2018 (35.4%) and 2019–2020 (36.4%; *p* = 0.45).

**Table 1:** Characteristics of current vape users and never-vapers in CSTS 2017–2018 and CSTS 2019–2020 (weighted)

	Current vape users		Students who never used vapes	
	2017–2018	2019–2020	2017–2018	2019–2020
	*n* = 11 966	*n* = 11 767	*n* = 105 971	*n* = 131 798
	% (95% CI)	% (95% CI)	% (95% CI)	% (95% CI)
Grade				
8	12.6 (9.1–16.1)	18.2 (12.6–23.7)	37.4 (31.1–43.7)	36.4 (33.7–39.0)
10	35.4 (33.5–37.2)	34.2 (31.5–36.9)	34.4 (30.9–37.9)	34.3 (32.8–35.7)
12	52.0 (49.5–54.5)	47.6 (44.1–51.2)	28.2 (25.4–31.1)	29.4 (28.1–30.6)
Gender				
Male	43.0 (41.7–44.2)	39.6 (37.3–41.8)	44.1 (43.4–44.8)	46.2 (45.7–46.8)
Female	41.7 (40.6–42.8)	48.7 (46.8–50.5)	47.6 (47.1–48.2)	47.2 (46.6–47.7)
Other	4.4 (3.9–4.9)	5.5 (4.7–6.4)	2.4 (2.2–2.6)	2.7 (2.5–2.9)
Declined to state/missing	10.9 (10.1–11.7)	6.2 (5.6–6.9)	5.9 (5.5–6.3)	3.9 (3.6–4.2)
Race and ethnicity				
NH-White	26.4 (24.1–28.7)	29.4 (26.4–32.5)	14.9 (13.5–16.4)	17.7 (15.4–19.9)
NH-Black	1.9 (1.6–2.3)	2.3 (1.8–2.7)	2.6 (2.3–2.9)	3.4 (2.9–3.9)
Hispanic	42.2 (39.7–44.7)	44.2 (41.6–46.8)	51.7 (49.2–54.3)	54.0 (50.9–57.1)
NH-Asian	6.0 (4.9–7.1)	6.8 (5.6–8.0)	11.6 (10.0–13.2)	11.3 (9.6–12.9)
NH-other	2.9 (2.5–3.2)	4.7 (4.0–5.5)	2.8 (2.5–3.1)	3.9 (3.6–4.2)
NH-multiracial	9.6 (8.9–10.3)	10.0 (8.9–11.1)	8.5 (7.9–9.1)	8.5 (7.7–9.2)
Declined to state/missing	11.1 (10.2–11.9)	2.7 (2.3–3.2)	7.9 (7.4–8.3)	1.4 (1.1–1.6)
Race and ethnicity (excluding declined to state/missing)				
NH-White	29.7 (27.2–32.2)	30.3 (27.2–33.3)	16.2 (14.6–17.8)	17.9 (15.6–20.2)
NH-Black	2.2 (1.8–2.5)	2.3 (1.9–2.8)	2.8 (2.5–3.2)	3.4 (2.9–3.9)
Hispanic	47.5 (44.7–50.3)	45.4 (42.7–48.1)	56.2 (53.3–59.0)	54.8 (51.6–57.9)
NH-Asian	6.8 (5.5–8.0)	7.0 (5.7–8.2)	12.6 (10.9–14.3)	11.4 (9.8–13.1)
NH-other	3.1 (2.7–3.5)	4.7 (4.0–5.5)	3.0 (2.7–3.3)	3.9 (3.6–4.2)
NH-multiple race	10.8 (10.0–11.6)	10.3 (9.1–11.4)	9.2 (8.6–9.8)	8.6 (7.9–9.3)
Days vaping in last 30 days				
1–9	64.6 (62.8–66.3)	63.4 (61.7–65.5)	–	–
10+	35.4 (33.7–37.2)	36.4 (34.5–38.3)	–	–

*CSTS *California Student Tobacco Survey, *CI *confidence interval, *EVALI *e-cigarette or vaping product use-associated lung injury, *NH *non-Hispanic

**Fig. 2:**
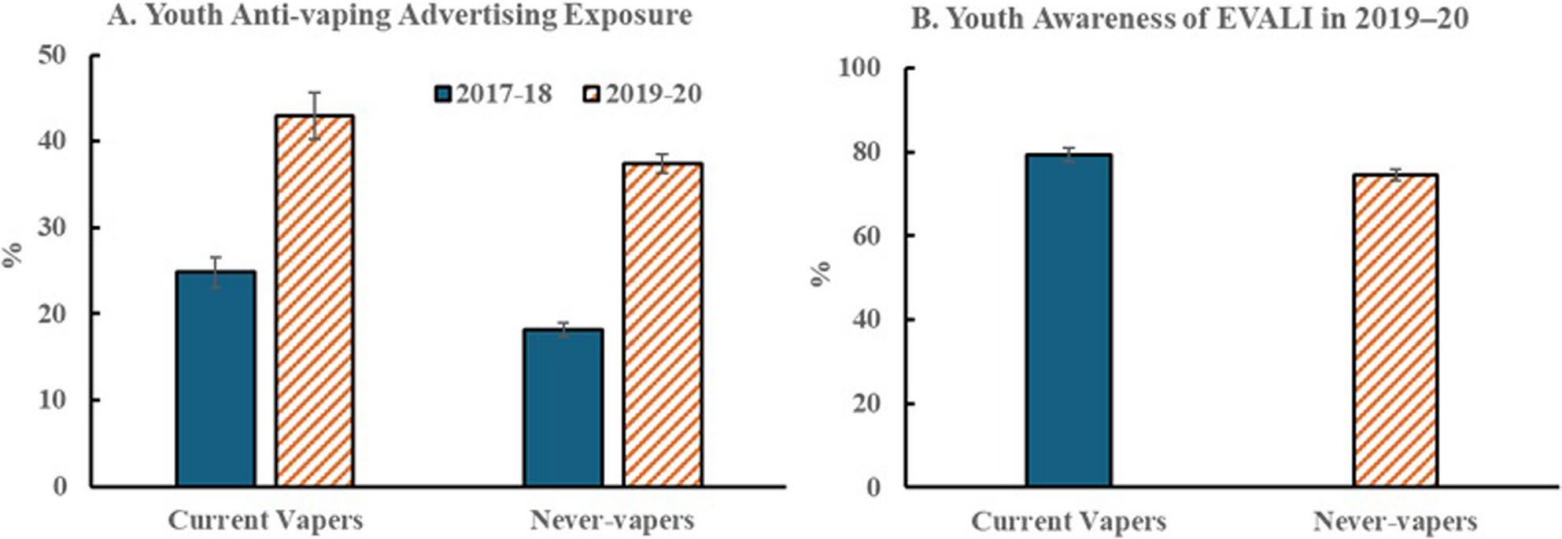
Youth anti-vaping advertising exposure and EVALI awareness in California. Panel A. Youth anti-vaping advertising exposure by vape use status. Panel B. Youth awareness of EVALI in 2019–20 by vape use status. Data source: California Student Tobacco Survey (CSTS) 2017–2018 and CSTS 2019–2020

Figure 2 shows the weighted distribution of participants by exposure to media messaging. Anti-vaping advertising exposure among students was significantly higher in 2019–2020 than in 2017–2018 for both current vape users and never-vapers (Panel A, all p’s < 0.001). EVALI awareness, only assessed in the later survey, was high in both groups (Panel B).

**Fig. 3:**
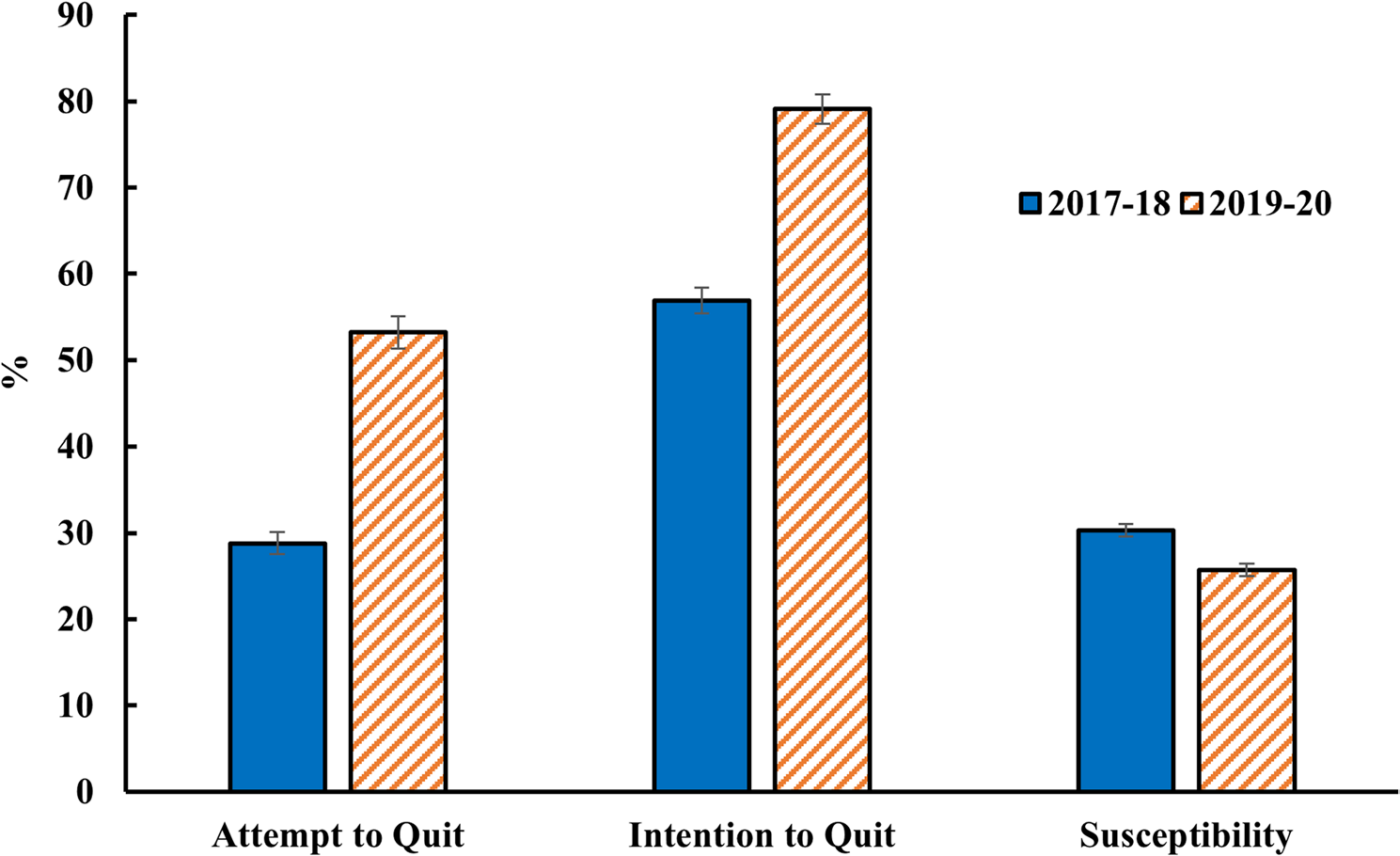
Youth attempts to quit vaping, intentions to quit, and susceptibility to future vaping. Note: Attempts and intentions to quit were assessed for current vape users. Susceptibility was estimated for never-vape-users. Data source: California Student Tobacco Survey (CSTS) 2017–2018 and CSTS 2019–2020

Figure 3 shows rates of attempting to quit and intending to quit among current vape users and susceptibility to future vaping among never-vapers. The rate of attempting to quit among current vape users was 53.2% in 2019–2020, significantly higher than 28.8% in 2017–2018 (*p* < 0.001); and the rate of intending to quit was also higher in 2019-20 than in 2017–18 (79.1% vs. 56.9%, *p* < 0.001). Among never-vapers, susceptibility to future vaping was lower in 2019-20 (25.7%) compared to in 2017–18 (30.3%,*p* < 0.001).

Table 2 shows the results of multivariable logistic regression analyses of the 2019–2020 data, controlling for demographics and tobacco use behaviors. Current vape users exposed to anti-vaping advertising were more likely to have attempted to quit than those not exposed (adjusted odds ratio [AOR] = 1.13; 95% CI: 1.01 to 1.28). Current vape users who had heard about EVALI were also more likely to attempt quitting than those who had not (AOR = 1.22; 95% CI: 1.06 to 1.42). Covariates negatively associated with attempting to quit included using other tobacco products and being offered vapes.

**Table 2:** Factors predicting quit attempts, intentions to quit, and susceptibility to future vaping, CSTS 2019–2020

	Attempted to quit vaping in past 12 months (*n* = 10 942)	Intended to quit vaping (*n* = 10 942)	Susceptible to future vaping (*n* = 127 142)
	AOR (95% CI)	AOR (95% CI)	AOR (95% CI)
Exposed to anti-vaping ads			
No	Ref	Ref	Ref
Yes	1.13 (1.01–1.28)	1.40 (1.16–1.69)	1.02 (0.97–1.07)
Heard about EVALI			
No	Ref	Ref	Ref
Yes	1.22 (1.06–1.42)	1.75 (1.47–2.08)	0.74 (0.69–0.78)
Grade			
8	Ref	Ref	Ref
10	1.23 (0.90–1.70)	1.70 (1.24–2.32)	0.86 (0.78–0.95)
12	1.01 (0.75–1.36)	1.88 (1.37–2.57)	0.85 (0.76–0.94)
Gender			
Male	Ref	Ref	Ref
Female	1.01 (0.90–1.15)	0.99 (0.80–1.22)	1.30 (1.23–1.37)
Other	0.91 (0.71–1.15)	0.51 (0.38–0.69)	1.37 (1.18–1.58)
Declined to state/missing	1.47 (1.00–2.16)	0.70 (0.46–1.05)	0.98 (0.86–1.11)
Race and ethnicity			
NH-White	Ref	Ref	Ref
NH-Black	1.08 (0.72–1.62)	1.44 (0.88–2.35)	0.78 (0.67–0.92)
Hispanic	0.90 (0.73–1.10)	0.81 (0.67–0.97)	1.25 (1.17–1.34)
NH-Asian	1.06 (0.87–1.28)	0.90 (0.64–1.27)	0.88 (0.80–0.97)
NH-other	1.17 (0.80–1.71)	0.83 (0.56–1.24)	0.80 (0.68–0.93)
NH-multiracial	0.91 (0.74–1.12)	0.70 (0.54–0.90)	1.00 (0.91–1.11)
Declined to state/missing	0.41 (0.17–0.97)	3.15 (1.17–8.47)	0.62 (0.33–1.17)
Parental education			
Not a college graduate	Ref	Ref	Ref
College graduate	0.99 (0.86–1.15)	0.97 (0.80–1.17)	0.94 (0.88–1.00)
Unknown/missing	1.20 (0.93–1.55)	1.12 (0.81–1.55)	0.88 (0.80–0.96)
Mental health status			
Good-to-excellent	Ref	Ref	Ref
Fair-to-poor	0.97 (0.84–1.13)	0.78 (0.64–0.94)	1.65 (1.56–1.75)
Days vaping in last 30 days			
1–9	Ref	Ref	–
10+	1.09 (0.95–1.25)	0.63 (0.53–0.74)	–
Use other tobacco products			
No	Ref	Ref	Ref
Yes	0.80 (0.68–0.95)	0.47 (0.39–0.57)	2.59 (2.06–3.25)
Offered vapes			
No	Ref	Ref	Ref
Yes	0.72 (0.59–0.88)	0.51 (0.39–0.66)	2.36 (2.24–2.49)

AOR=adjusted odds ratio; CI=confidence interval; CSTS=California Student Tobacco Survey; EVALI=e-cigarette or vaping product use-associated lung injury; NH=non-Hispanic; Ref=reference group. Quit attempts and intentions to quit were assessed for current vape users. Susceptibility was estimated for never-vapers

The patterns for current vape users’ intentions to quit were similar to those for quit attempts. Exposure to anti-vaping advertising was positively associated with intending to quit (AOR = 1.40; 95% CI: 1.16 to 1.69), as was awareness of EVALI (AOR = 1.75; 95% CI: 1.47 to 2.08). Covariates negatively associated with quitting intentions included having a gender minority status, being Hispanic or multiracial, having fair-to-poor mental health, vaping on 10 or more days in the last month, using other tobacco products, and being offered vapes. Being older (grade 10 or 12) and not reporting race/ethnicity were positively associated with quitting intentions.

The patterns for susceptibility to future vaping among never-vapers were somewhat different. No significant association was found between exposure to anti-vaping advertising and susceptibility to future vaping (AOR = 1.02; 95% CI: 0.97 to 1.07). EVALI awareness, however, was a protective factor against future vaping (AOR = 0.74; 95% CI: 0.69 to 0.78). Being female or of sexual minority status, having fair-to-poor mental health, using other tobacco products, and being offered vapes were positively associated with susceptibility to future vaping. Being older (grade 10 or 12) was negatively associated with susceptibility. Relative to White students, Hispanic students had greater susceptibility while Black and Asian students and those of other races were less susceptible.

Supplemental Table 1, for readers who are interested, shows the rates behind the odds ratios in Table 2.

## Discussion

This study, based on two cycles of a large population survey of adolescents in California conducted before the COVID-19 pandemic, showed that a significantly higher proportion of current vape users in 2019–20 had attempted to quit than those in 2017–18. A greater proportion of vape users in 2019–20 intended to quit than those in 2017–18. There was also a significantly lower proportion of students who never vaped intended to try vaping in 2019–20 than in 2017–18. Exposure to anti-vaping advertising and awareness of EVALI were found to be associated with higher odds of quit attempts and intentions to quit. Awareness of EVALI was also associated with lower odds of susceptibility to future vaping among adolescents who had never vaped.

Previous research has investigated the effects of anti-vaping advertising on quit attempts and reported positive associations. However, the results have not yet reached statistical significance, possibly due to the small sample sizes and limited statistical power [30]. To our knowledge, no study has examined the effects of EVALI awareness on quit attempts among adolescents. Previous research has also examined the link between anti-vaping advertising and intentions to quit among adolescents who vape [30, 31]. Two studies found that awareness of EVALI was associated with a greater risk perception of vaping [8, 22]. One showed that the impact of EVALI on quitting intentions may be mediated via risk perception [22]. To our knowledge, the present study is the first to examine the relationship between anti-vaping advertising, EVALI awareness, and quit attempts in a single regression analysis. It found that anti-vaping advertising and EVALI awareness each predicted quit attempts when controlling for the effect of the other. Each also predicted intentions to quit in the multivariable analysis.

Prominent anti-vaping advertising campaigns and media reports on EVALI all conveyed negative messages about vaping and appeared around the same time, in 2018–2019. The multivariable regression analysis in this study, therefore, adds some precision in estimating the unique contributions of each in a statistical sense.

The study found that exposure to anti-vaping advertising was not associated with a lower odds of susceptibility to future vaping among adolescents who had never vaped. Two population-based studies found similar results [32, 33], while an experimental study showed that such advertising significantly lessened susceptibility [34]. The study found, however, that awareness of EVALI was significantly associated with lower susceptibility to future vaping.

Media reports on EVALI were generally based on press releases from recognized health authorities, such as the Centers for Disease Control and Prevention [18], which often included cumulative numbers of hospitalizations and deaths associated with the outbreak. Reports sometimes included stories of individual patients suffering from EVALI. Moreover, media coverage of the outbreak was extensive. It lasted more than six months, a long time compared to coverage of other public health events, such as outbreaks of food poisoning [35]. The current study found that media coverage of EVALI had significant effects on adolescents’ cognition and behavior, even though the reports were often inaccurate about the cause of EVALI [8, 16, 18]. In the study sample, EVALI awareness actually had more consistent predictive value than anti-vaping advertising (Table 2), a remarkable finding considering that the two national media campaigns had combined expenditures exceeding $100 million annually [8], whereas EVALI awareness came about primarily through earned media.

The regression analysis of the 2019–2020 CSTS data is limited because we could not translate correlational statistics directly into estimates of how much the media campaigns and EVALI reports caused students to attempt quitting, intend to quit, or become less susceptible to vaping in the future. Moreover, regression analysis cannot account for the potential indirect effects of anti-vaping advertising exposure. For example, the diffusion of media messages among peers may influence individuals who were not directly exposed, leading to an underestimation of the true impact of media interventions [36]. The study does, however, provide a comparison of quit attempts, quitting intentions, and susceptibility between 2017–2018 and 2019–2020. The difference in quit attempts was dramatic, nearly doubling (Fig. 3). This, too, was remarkable, as quit attempt rates generally do not change much on a population level [37, 38]. The difference in quit intentions was also large. The difference in susceptibility was less dramatic, but never-vapers account for a much larger segment of the adolescent population. Small changes in their susceptibility to future vaping could have significant impacts on overall vaping prevalence.

These large differences between the two periods cannot be solely attributed to media, as there were many contemporaneous tobacco control activities taking place [39–42]. Even so, the regression analysis provides clues about two important contributing factors, suggesting that aggressive anti-vaping advertising campaigns and media coverage of EVALI were both influential on adolescents.

These changes in California may offer insight into mechanisms relevant to other jurisdictions in the U.S. that experienced similar media environments and policy contexts. One would predict a significant drop in adolescent vaping prevalence post-2019. Moreover, the dramatic differences shown in Fig. 3 help explain why 2019 became an inflection point. The prevalence of vaping among U.S. adolescents has continued to decline since then (Fig. 1). It is worth noting that this trend contrasts with what has been observed in England, where vaping among youth has continued to increase after a brief dip in 2021 during the COVID-19 pandemic [43]. Further examining the underlying factors and approaches adopted by different countries to address adolescent vaping could provide valuable insights for future tobacco control strategies.

This study has limitations. First, there are limitations in measurement. The measure of advertising exposure was based on only two questions. The susceptibility measure was shortened from the original scale [21]. In assessing EVALI awareness, the survey item did not explicitly use the term EVALI. As a result, the measure may capture exposure to broader negative information about vaping. There is a concern of conceptual overlap between EVALI awareness and anti-vaping advertising exposure. However, as mentioned earlier in the method section, sensitivity analyses including both measures simultaneously and independently yielded stable coefficients for both EVALI awareness and anti-vaping advertising exposure, without evidence of coefficient instability or sign reversal, suggesting that any overlap did not significantly bias model estimates. Second, there was a terminology change of vapes from “e-cigarettes” in 2017–18 to “nicotine vapes” in 2019–20. It is possible that a small number of cannabis-only vape users may have self-identified as e-cigarette users in the 2017–18 survey. However, given that the magnitude of differences in quit attempts and intentions to quit between 2017–2018 and 2019–2020 was so large, it is unlikely that any misclassification has significantly affected the study’s conclusions. Third, the study used results from state surveys to gain some insights into the national trend in adolescent vaping because no national survey assessed both exposure to anti-vaping advertising and EVALI awareness. The lack of a direct measure of EVALI awareness from a national survey of adolescents naturally limits the generalizability of these results. Finally, the California surveys were conducted biannually, meaning there were no prevalence data for the 2018–19 school year, which national surveys show to be an inflection point in adolescent vaping prevalence. However, the 2017–2018 and 2019–2020 CSTS data do show a decline from 10.5% to 8.2% [19, 20], consistent with the national trend (Fig. 1).

## Conclusions

The year 2019 was a clear inflection point in the vaping trend among U.S. adolescents, a phenomenon that seems to have not received sufficient attention from the research community. This study suggests that the dramatic news coverage of EVALI of that year and the aggressive anti-vaping advertising that started a year earlier may have played significant roles in the changing vaping-related cognition and behavior among U.S. adolescents. Further research to better understand the causes of these changes could help solidify the public health gains by ensuring that vaping among adolescents continues to decline, just as cigarette smoking has [2]. 

## Supplementary Information

Supplementary Material 1.

## Data Availability

Data from the California Student Tobacco Survey (CSTS) are owned by the California Department of Public Health. The data are available to interested researchers upon request. For CSTS data, please contact Dr. Xueying Zhang of the California Department of Public Health at ( [Xueying.Zhang@cdph.ca.gov](mailto: Xueying.Zhang@cdph.ca.gov) ).

## Abbreviations

AOR: Adjusted odds ratio
CI: Confidence interval
CSTS: California Student Tobacco Survey
EVALI: E-cigarette or vaping use-associated lung injury
FDA: Food and Drug Administration
NH: Non-Hispanic
THC: Tetrahydrocannabinol
