# Supplementary material for: EVALI, anti-vaping advertising, quit attempts, and susceptibility: insights into the 2019 inflection point for U.S. adolescent vaping from California data
Source: BMC Public Health. 2026 Apr 2;26:1353. doi: 10.1186/s12889-026-27209-3 (PMC13107825; doi:10.1186/s12889-026-27209-3)
Supplement: Supplementary file 1 — Supplementary Material 1. [file 12889_2026_27209_MOESM1_ESM.docx]

**SUPPLEMENTAL TABLE 1.** Youth quit attempts, intentions to quit, and susceptibility to future vaping, CSTS 2019–2020

|  | Attempted to quit vaping in past 12 months  (n=10 942) | Intended to quit vaping  (n=10 942) | Susceptible to future vaping  (n=127 142) |
| --- | --- | --- | --- |
|  | % (95% CI) | % (95% CI) | % (95% CI) |
| Exposed to anti-vaping ads |  |  |  |
| No | 52.1 (49.9–54.3) | 76.0 (73.8–78.1) | 25.7 (24.9–26.4) |
| Yes | 54.5 (52.0–57.0) | 83.8 (81.6–86.0) | 25.7 (24.6–26.8) |
| Heard about EVALI |  |  |  |
| No | 49.5 (45.6–53.5) | 69.4 (66.3–72.4) | 29.4 (28.3–30.5) |
| Yes | 54.2 (52.4–55.9) | 81.9 (80.4–83.5) | 24.5 (23.6–25.3) |
| Grade |  |  |  |
| 8 | 51.9 (44.5–59.4) | 70.7 (64.4–77.0) | 25.8 (23.8–27.7) |
| 10 | 55.6 (52.9–58.2) | 79.6 (77.7–81.5) | 25.8 (25.2–26.3) |
| 12 | 51.9 (50.4–53.4) | 81.9 (80.5–83.2) | 25.7 (25.0–26.4) |
| Gender |  |  |  |
| Male | 52.8 (50.4–55.3) | 80.1 (77.9–82.3) | 22.1 (21.3–23.0) |
| Female | 53.2 (50.7–55.7) | 81.5 (78.8–84.1) | 28.9 (27.8–29.9) |
| Other | 51.7 (46.0–57.4) | 59.8 (50.9–68.7) | 33.9 (30.8–37.0) |
| Declined to state/missing | 56.1 (50.5–61.6) | 70.8 (66.1–75.4) | 24.8 (23.0–26.5) |
| Race and ethnicity |  |  |  |
| NH-White | 54.2 (51.2–57.2) | 82.6 (79.9–85.4) | 23.5 (22.2–24.8) |
| NH-Black | 55.7 (45.6–65.8) | 84.1 (78.8–89.4) | 20.2 (18.0–22.4) |
| Hispanic | 51.7 (48.8–54.6) | 77.7 (75.5–79.9) | 28.5 (27.7–29.4) |
| NH-Asian | 56.2 (52.4–60.0) | 83.2 (79.1–87.3) | 20.5 (19.1–21.9) |
| NH-other | 58.3 (50.3–66.3) | 72.9 (65.6–80.2) | 20.5 (18.3–22.6) |
| NH-multiracial | 51.9 (47.1–56.7) | 74.5 (69.3–79.6) | 24.3 (22.6–26.0) |
| Declined to state/missing | 51.8 (45.7–57.9) | 75.6 (70.5–80.7) | 23.7 (20.2–27.2) |
| Parental education |  |  |  |
| Not a college graduate | 52.2 (49.8–54.7) | 79.4 (77.3–81.4) | 28.4 (27.6–29.3) |
| College graduate | 52.8 (50.5–55.1) | 80.2 (77.7–82.8) | 24.0 (22.9–25.1) |
| Unknown/missing | 56.5 (51.4–61.7) | 74.7 (70.1–79.2) | 24.2 (22.9–25.5) |
| Mental health status |  |  |  |
| Good-to-excellent | 53.3 (51.2–55.5) | 81.4 (79.3–83.5) | 22.4 (21.5–23.3) |
| Fair-to-poor | 52.8 (49.7–55.9) | 76.8 (74.5–79.0) | 34.2 (33.2–35.2) |
| Days vaping in last 30 days |  |  |  |
| 1–9 | 53.3 (50.9–55.6) | 83.4 (81.5–85.3) | – |
| 10+ | 52.9 (50.5–55.3) | 71.5 (68.8–74.2) | – |
| Use other tobacco products |  |  |  |
| No | 54.5 (52.4–56.6) | 84.0 (82.4–85.6) | 25.5 (24.7–26.3) |
| Yes | 49.4 (45.9–52.9) | 66.1 (63.0–69.2) | 51.0 (46.4–55.6) |
| Offered vapes |  |  |  |
| No | 58.8 (55.0–62.7) | 86.9 (84.4–89.3) | 22.4 (21.6–23.2) |
| Yes | 51.9 (49.9–53.9) | 77.6 (75.8–79.5) | 41.6 (40.5–42.7) |

Note: CI=confidence interval; CSTS=California Student Tobacco Survey; EVALI=e-cigarette or vaping product use-associated lung injury; NH=non-Hispanic. Quit attempts and intentions to quit were assessed for current vapers. Susceptibility was estimated for never-vapers.
